# Refining Climate Change Projections for Organisms with Low Dispersal Abilities: A Case Study of the Caspian Whip Snake

**DOI:** 10.1371/journal.pone.0091994

**Published:** 2014-03-26

**Authors:** Tiberiu C. Sahlean, Iulian Gherghel, Monica Papeş, Alexandru Strugariu, Ştefan R. Zamfirescu

**Affiliations:** 1 Faculty of Biology, University of Bucharest, Bucharest, Romania; 2 Department of Terrestrial Fauna, “Grigore Antipa” National Museum of Natural History, Bucharest, Romania; 3 Department of Zoology, Oklahoma State University, Stillwater, Oklahoma, United States of America; 4 Faculty of Biology, “Alexandru Ioan Cuza” University, Iaşi, Romania; The Ohio State University, United States of America

## Abstract

Climate warming is one of the most important threats to biodiversity. Ectothermic organisms such as amphibians and reptiles are especially vulnerable as climatic conditions affect them directly. Ecological niche models (ENMs) are increasingly popular in ecological studies, but several drawbacks exist, including the limited ability to account for the dispersal potential of the species. In this study, we use ENMs to explore the impact of global climate change on the Caspian whip snake (*Dolichophis caspius*) as model for organisms with low dispersal abilities and to quantify dispersal to novel areas using GIS techniques. Models generated using Maxent 3.3.3 k and GARP for current distribution were projected on future climatic scenarios. A cost-distance analysis was run in ArcGIS 10 using geomorphological features, ecological conditions, and human footprint as “costs” to dispersal of the species to obtain a Maximum Dispersal Range (MDR) estimate. All models developed were statistically significant (*P*<0.05) and recovered the currently known distribution of *D. caspius*. Models projected on future climatic conditions using Maxent predicted a doubling of suitable climatic area, while GARP predicted a more conservative expansion. Both models agreed on an expansion of suitable area northwards, with minor decreases at the southern distribution limit. The MDR area calculated using the Maxent model represented a third of the total area of the projected model. The MDR based on GARP models recovered only about 20% of the total area of the projected model. Thus, incorporating measures of species’ dispersal abilities greatly reduced estimated area of potential future distributions.

## Introduction

Climate is recognized as one of the main factors responsible for shaping large-scale species distributions [1]. Global climate change observed over the past decades has produced shifts in the distribution and abundance of numerous species [2], [3] and is responsible for species extinction [4]. Increased levels of global warming are expected to have different effects on species, based on their life-history traits [5], [6], dispersal rates [7]–[9], and habitat requirements [10]–[12]. Predicted outcomes include range shifts following changing environments, adaptation to novel conditions, isolation to unaffected regions (refugia), and extinction [2], [13]–[16].

Ectothermic animals (such as amphibians and reptiles) are especially prone to be affected by global warming as a result of their particular ecological requirements [17]. Local extinctions are well documented for reptiles [18]–[21] and amphibians have the highest rate of extinction recorded to date [22]–[27]. For example, Sinervo et al. [28] predicted that by 2080, 30% of all known lizard species will be locally extinct due of global warming alone. In turtles, Ihlow et al. [29] calculated that changing climates will produce distribution reductions of 86% of all species and 12% of the species will shift completely out of their current range. In snakes, Reading et al. [30] found that, in a period of about 14 years, 11 of 17 populations across Europe, Africa, and Australia have faced abrupt declines. Reading et al. [30] suggested that the reasons for these declines are the same as for the ones observed in other reptile groups but with a special emphasis on global climate change as the root cause.

Ecological niche modeling has become an increasingly popular methodology to study species’ potential distributions in recent years and, as a result, several applications have been developed to facilitate generation of such models and distributions [31]–[34]. Broadly, these tools differ with regards to the type of species’ records (presence/absence or presence-only) and predictors utilized (climatic - empirical approach or physiological constrains - mechanistic approach) [35]–[37]. In their current form, ecological niche models (ENMs) allow us to infer present [38]–[41], past [42], [43], and future potential distribution of species [44]–[46], speciation scenarios [47]–[50], to design or refine protected areas for threatened species [51], [52], and to predict novel distributions of invasive taxa [53], [54] and impact of human-mediated global warming on wildlife [55]–[57].

As stated, ecological niche modeling is a popular approach to assessing the impact of global climate changes on species’ ecological niches [53], [56], [58]–[60]. However, resulting predictions of future distributions are sometimes prone to potentially erroneous interpretations if the differences in dispersal ability among taxa are not taken into account. As such, the interpretation of these predictions may be subjective, indicating either species’ expansion or contraction of their current ranges, or complete relocation to a new geographic area where the climate remains favorable. Furthermore, if the climatic change occurs rapidly, there is a risk of extinction for certain species, given the concept of niche conservatism, which states that species and clades retain their niches and related ecological traits over time [61], [62].

One of the main drawbacks of ecological niche modeling, in its empirical implementation, is its reliance on the correlation between abiotic factors (i.e. temperature, humidity, radiation) and species’ presences in what is defined as a “bioclimatic envelope” [46], [63]. Other factors are known to play an important role in the distribution of species, such as biotic interactions (i.e. predators, prey, diseases) [64], and dispersal ability or dynamics of vegetation [65]. In the context of climatic shifts, dispersal ability stands out as one of the most important factors not taken into account when generating estimates of species’ distributions. It is generally futile to discuss the suitability of future climatic conditions in a certain area that the species may not be able to reach in the first place.

In the present study, we used (i) ENMs to estimate the impact of climate change scenarios on the geographic extent of a narrow range, low dispersal organism (*Dolichophis caspius*) and (ii) cost distance analysis in Geographic Information Systems (GIS) to assess species’ dispersal potential into novel, currently unoccupied regions identified as suitable by ENMs. Limitations of this approach are also discussed. Our main goal was to develop an approach to adjust assessments of global warming effects on narrow range, low dispersal organisms such as the Caspian whip snake by incorporating spatial estimates of the species’ dispersal capacity on the landscape.

## Materials and Methods

### Species Account

The Caspian whip snake (*Dolichophis caspius*) is a xerophilous snake species inhabiting primarily steppe open grasslands, Mediterranean scrublands, rocky outcrops, and broad-leaved forest edges at low and medium altitudes (0 to 1600 m ASL) [66], [67]. The species is widespread in Eastern Europe, southern Ukraine, the Balkan Peninsula, West Anatolia, Black Sea Coast, east to the Caucasus Mountains, southern Russia, and Kazakhstan [66]–[68]. In Eastern Europe (Romania, Bulgaria, Greece) the species is possibly one of the most frequent victims of ever-increasing road traffic and its habitat is experiencing ongoing reduction in many parts of the range [69].

Nagy and collaborators [70] recognized two Caspian whip snake haplotype groups separated by the Aegean Sea and the Bosphorus Straight, and estimated to have diverged during the Pleistocene: an eastern group along the Turkish coast and on East Aegean islands, and a western group in the Cyclades islands, Euboea island, and mainland Central and Eastern Europe. The western haplotype may have survived in its current observed range during glacial periods and persisted since, but later rapid recolonization events of Central and southeastern Europe, very likely from the Balkan Peninsula, represents another possible explanation of the current range [70].

Lowland areas such as steppes, forest-steppes, and xeric forests, the preferred habitats for the Caspian whip snake [66]–[68], are especially fragile and prone to land use changes due to their value as agricultural and grazing fields. In addition, these areas are extremely sensitive to minor variations in humidity and temperature, i.e. to the effects of climate change [71]–[74]. In the European Union (EU), such ecosystems became a top priority for conservation (EU Habitats Directive 92/43/EEC of 21 May 1992), but in developing countries conservation measures are implemented at a slower pace and may not represent a priority. At the same time, most of the range of *Dolichophis caspius* falls outside of the EU, where such measures are limited or do not exist [75].

### Species Occurrence Data

In order to maximize the quantity and quality of the occurrence data used for generating the models, we did an extensive literature review from which we extracted available geographic location information. Imprecise localities such as country, county, coarse resolution UTM grids, as well as locations with uncertainty higher than approximately1 km were excluded from the analysis. Most of the whip snake localities had more than one confirmed record in the past 50 years. The extracted points were manually georeferenced using ArcGIS 10 with populated places and topographic maps as reference layers. For locality descriptions that could not be georeferenced, we contacted the authors to clarify the geographic reference. The georeferenced dataset comprised 338 localities which we further trimmed to retain only spatially unique ones, corresponding to single environmental grid cells using Trim Data function in ENMTools 1.3 [76]. Consequently, only 324 unique records were used to generate ENMs for the Caspian whip snake (Supporting Information S1).

### Climate Data

The baseline (current) climatic data used for running the models had a spatial resolution of 30 arc-seconds (approximately 1 km) and was retrieved from the WorldClim database [77], [78]. To analyze future climate effects on the potential distribution of the whip snake, we used future climate datasets produced by the Canadian Centre for Climate Modeling and Analysis (CCCma) using the Second Generation Coupled Global Climate Model (CGCM2), for two greenhouse gas emissions scenarios, A2a and B2a. All datasets were downloaded at 2.5 arc-minutes resolution (4.5 kilometers) from the International Centre for Tropical Agriculture website [79]. The chosen emission scenarios follow two opposite views on how the climate will change in the future 70 years. The A2a scenario is considered more liberal and takes into account a high population growth worldwide, increased energy use, land-use changes, and a slow technological advance; the B2a scenario is considered more conservative and simulates a slow human population growth rate, limited land use changes, and reflects a more technologically innovative world [80]. For each scenario we used the projections for 2020, 2050, and 2080 in an effort to forecast time series changes in the climatic niche distribution of *D. caspius*.

Both climatic datasets (baseline and CGCM2) comprise 19 bioclimatic variables (see Hijmans et al. [78] for more details) (Table 1) considered to compute more robust models than monthly temperature and precipitation variables [81] also available in the datasets [78]. However, to obtain robust models, it is necessary to optimize variable use by avoiding highly correlated variables or by selecting those that are increasing model accuracy. Here we opted for the latter to select a subset of the available 19 variables. We ran a first model using all 19 variables (Table 1) in Maxent, selecting for the second and final model runs only the variables that had a contribution above 5% in creating the first model (Table1). The same subset of variables was used for generating the GARP models.

**Table 1:** Variable selection results indicating percent contributions to the initial and last models.

	*Contribution*	
*Variable*	First model	Final model
Mean Temperature of Coldest Quarter	35.2	39.7
Temperature Seasonality	17.1	21.6
Mean Diurnal Range	12.7	16.1
Min Temperature of Coldest Month	6.4	12.3
Precipitation of Driest Month	5	10.3
Temperature Annual Range	4.8*	–
Mean Temperature of Wettest Quarter	3.3*	–
Mean Temperature of Warmest Quarter	3.1*	–
Isothermality	2.8*	–
Precipitation of Driest Quarter	2.6*	–
Annual Mean Temperature	1.7*	–
Precipitation of Coldest Quarter	1.5*	–
Precipitation Seasonality	1.1*	–
Max Temperature of Warmest Month	0.6*	–
Mean Temperature of Driest Quarter	0.6*	–
Annual Precipitation	0.6*	–
Precipitation of Warmest Quarter	0.5*	–
Precipitation of Wettest Month	0.2*	–
Precipitation of Wettest Quarter	0.2*	–

*variables eliminated due to low contribution to model development.

To meet the recommendation that models must be trained only in a region within the known range of the species or within its dispersal limits [82], [83], we produced the model using as geographic extent only the known distribution range of the *D. caspius*, following the distributional limits given in the literature [66].

### Ecological Niche Modeling Procedure

Based on the species records and the bioclimatic variables, we generated models using Maxent version 3.3.3 k [36] and Desktop GARP (Genetic Algorithm for Rule-set Prediction) version 1.1.3 [84]. These two algorithms are among the ones designed for presence-only datasets that produce reliable predictions [85]. Also, previous studies [86]–[88] have shown that GARP tends to produce wider potential distributions when compared with Maxent, thus we employed both algorithms to assess degree of variation in the potential distribution and the estimated impact of global warming on the species studied.

GARP is a machine-learning algorithm, while Maxent has recently been reclassified as a version of the generalized linear model [89]. Both algorithms are able to produce ecological niche models using presence-only data and environmental predictors [36], [90]. Maxent does this by finding the distribution closest to uniform distribution (maximum entropy) constrained by the environmental data input [36]. On the other hand, GARP generates models using rules that are applied to the training data. The changes in the predictive performance between runs are used to evaluate whether a rule is included in a model [32].

To produce the models in Maxent we used the default settings including regularization multiplier = 1 and maximum number of background points = 10,000. Random test percentage was set to 25% of the input species’ occurrence records to test the performance of the resulting model. Also, the clamping option was used to downweight areas outside of the range presented by the training data (Supporting Information S2). The clamping function produces a map output that identifies in the projections (i.e., future climate) areas with environmental variable values outside of the minimum and maximum range of values present in the training region (i.e., present-day climate). Model predictions in such areas are deemed uncertain [91]. In GARP, we ran 100 models with a 0.01 convergence limit of model iterations and the maximum itineration number set to 1000. We also activated the internal testing feature and the “best subsets” procedure [92] to select ten best models as the final ones. The selection criteria included omission error (i.e., known occurrences predicted absent) which we set to the lowest 20% of values, and commission error (i.e., areas without known presences predicted present) for which we used the default 50% value. Both Maxent and GARP models were projected onto climate change scenario datasets at the end of the iteration phase. As the final procedure, in ArcGIS 10 (ESRI, Redlands, CA) [93] we summed the best ten GARP models to create a model agreement map and we converted Maxent and GARP models with continuous probability distribution values and model agreement values, respectively, to binary presence-absence potential distribution maps using as threshold 10% omission error of the training presence dataset. These post-modeling procedures were employed for both present-day and future climate potential distributions.

### Model Evaluation

The model evaluation was done using three different methods: (1) partial ROC (Receiver Operating Characteristic), (2) omission error calculated using the test occurrence subset and thresholded, presence-absence predictions, and (3) expert’s opinion. Generally, the most frequent method to evaluate ENMs is the Area Under the ROC Curve (AUC) [36], but, as it has been shown that this approach may not be the most adequate to evaluate the ecological niche models (see Peterson et al. [92], [94] for details), we used a modified version, the partial ROC [80], which calculates the AUC only for a portion of the ROC curve, above an omission error threshold. We set the threshold to 5%. The partial ROC AUC scores were calculated using the partialAUC application developed by N. Barve (University of Kansas). We ran 100 iterations in which the test occurrence data were bootstrapped and we used z tests to assess whether the partial ROC AUC values were above that of a random model.

### Post-modeling Analysis

To estimate whether *D. caspius* could disperse into novel areas of suitable climatic conditions predicted by the models, we used the cost distance analysis tool in ArcGIS 10. The cost distance function calculates the “effort” or “resistance” to moving from one point to another on the landscape based on a “cost” raster (GIS grid with cells, or pixels). If no destination point is given, the function automatically calculates “effort” to the edge of the raster. The cost raster is used to explain the difficulty of crossing certain landscape features, topographic or ecological (e.g.: altitude, slope, rivers, ecoregions, human-impacted areas etc.; Supporting Information S3–S5).

To develop the cost raster, we used geomorphological features (altitude and rivers), ecological conditions (ecoregions and presence-absence Maxent and GARP rasters), and the impact of human habitation on the landscape (human footprint). The altitude raster used was included in the standard WorldClim BIOCLIM package and was used to calculate a slope raster in ArcGIS 10. A GIS layer with world ecoregions was downloaded from WWF (http://worldwildlife.org), while the human footprint raster was downloaded from NASA Socioeconomic Data and Applications Center (SEDAC, http://sedac.ciesin.columbia.edu). River data were available in the basic collection of layers offered by ESRI with the license of ArcGIS 10. The above-mentioned rasters were reclassified manually using the “Reclassify” function in ArcGIS 10, while the number of classes varied depending on the specific raster dataset (Supporting Information S5, S6). The values for each interval were assigned based on species’ biology and experts’ opinion to account for the difficulty of movement (Supporting Information S3–S4). “Cost” rasters were created using the “Mosaic to New Raster” command in ArcGIS 10, which merged all reclassified rasters to a new dataset (Supporting Information S5–S6). To ensure that the cells of the resulting cost raster reflected the highest impediment to movement, priority was given to cells with a higher classification value (i.e., cells associated with higher costs). We generated separate cost rasters for GARP and Maxent model outputs and for each climate projection (two emission scenarios and three time periods; Supporting Information S3–S4). Also, to account for the uncertainty associated with assigning costs based on experts’ opinion, we developed three separate scenarios (based on three separate cost rasters; Supporting Information S3–S4): (S1) *a permissive scenario*, in which the populations were assumed to have higher capacity of dispersal, (S2) *a restrictive scenario*, where populations were heavily constrained by conditions outside of the known environmental range, and (S3) *a balanced scenario*, in which the cost values were weighted based on experts’ opinion of which environmental conditions are more likely to be suitable for the Caspian whip snake’s dispersal and which could impede movement through the landscape.

The cost rasters (Supporting Information S3–S4), along with the occurrence points (Supporting Information S1) gathered from the literature survey, were used as input in the cost distance analysis performed in ArcGIS 10, “Cost Distance” function, which calculates the cumulative cost value for each cell (pixel) on the landscape as an individual would disperse from species’ known presence localities. The rasters generated through the cost distance analysis (for both modeling algorithms and climatic scenarios, over all three time periods and dispersal scenarios) were then reduced (thresholded) using the lowest (minimum) cost distance value that connected all species’ known presences (Supporting Information S5–S6). The threshold was used since information regarding home range or dispersal capabilities for the Caspian whip snake is not available. We refer to the resulting rasters as Maximum Dispersal Range (MDR). The cost distance analysis was repeated for each cost raster generated for the two ecological niche modeling algorithms and two emission scenarios, for three time periods and three dispersal scenarios. We emphasize that, in addition to ENMs, MDR incorporates variables not directly used in the ecological niche modeling algorithms, such as anthropogenic alterations of the landscape and topography, that would affect dispersal routes for this species.

## Results

### Model Accuracy Metrics

**Figure 1:**
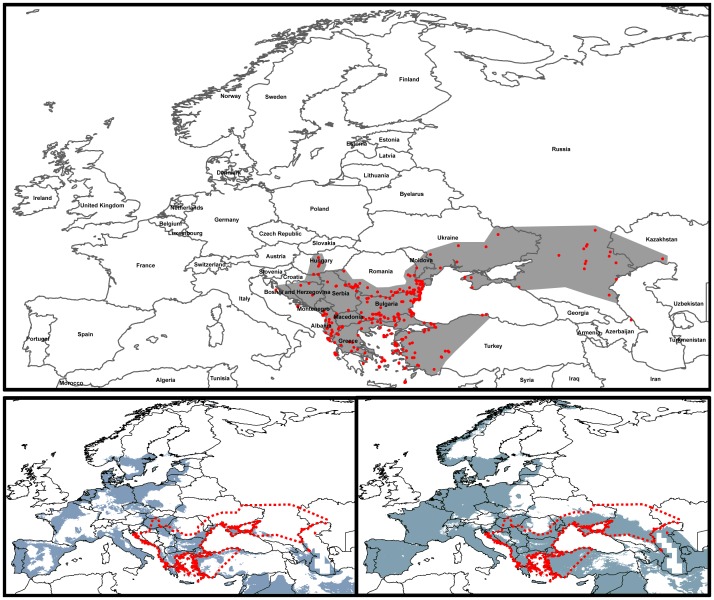
Present-day ecological niche models (green) for *D. caspius* (lower left - model generated by Maxent; lower right - model generated by GARP) in comparison to its known distribution range (upper panel, area in grey) (adapted after [66]) and presence records (red points). The red dotted line in lower panels corresponds to the grey area in upper panel (known distribution range).

The partial ROC area under the curve (AUC) ratios had a mean of 1.24 (SD = 0.064) for Maxent and a mean of 1.18 (SD = 0.06) for GARP in 100 replicates, and were statistically significant above the null expectations (z test, p<0.05). The rate of false negative records (omission error) was 13% of the total number of presences for Maxent model and 0% for GARP model. From the herpetologist expert’s point of view, both algorithms recovered the known distribution and potential distribution of the Caspian whip snake (Fig. 1), with a slight trend of Maxent to underpredict the potential distribution of Caspian whip snake in the northern part of the species’ range. The location of presence records predicted absent (omission error) is in agreement with the expert opinion, as these records are at the limits of the species’ distribution. The results from all three evaluation methods suggest good predictive power of the models, thus we consider the resulting species’ potential distributions under future climate conditions to be reliable estimates of the effects of forecasted climate change.

### Present-day Potential Distribution of the Caspian whip Snake

The highest contributing variables (>5%) to both the first and the final models were mean temperature of the coldest quarter, temperature seasonality, mean diurnal range, minimum temperature of the coldest month, and precipitation of the driest month (Table 1). The difference in variable contribution values between the two model runs was not significant (*t* = −0.622, *df = *8, *p* = 0.551). Maxent and GARP models predicted most of Balkan Peninsula, Pannonian Plain, Crimean Peninsula, and Western and Southern coast of Caspian Sea, Italy, Syria, Iraq and Iberian Peninsula as climatically suitable for *D. caspius*. Other climatically suitable regions were found in parts of Germany, Turkey, France, Poland, and Baltic and Scandinavian countries.

We observed disagreement in the present-day potential distributions predicted by the two algorithms: large areas predicted suitable only by GARP in Turkey, Romania (excluding the Carpathian Mountains), Moldova, Southern Ukraine, southern European Russia, and the Caspian Sea basin, including large parts of Turkmenistan, Azerbaijan, and Georgia, were only marginally predicted present by the Maxent model. In addition, according to the GARP present-day model, all Western Europe and the Czech Republic are climatically suitable for *D. caspius*, regions only partially predicted present by the Maxent model. Generally, discrepancies aside, the models generated by both GARP and Maxent produced pertinent maps of the potential distribution of Caspian whip snake under current climatic conditions.

Moreover, the GARP model recovered almost all of the species’ occurrence points used in the analysis (i.e, low omission error) and filled in the gaps in species range in the southern European Russia, Ukraine, and Moldova, where, although anecdotal information about the species’ presence exists, to the best of our knowledge, no documented records are available. From the herpetologist’ point of view (expert opinion), the prediction generated by GARP is a closer approximation of the current potential distribution of *D. caspius*, with a tendency to overpredict especially at the northern limit of the species’ range.

### Forecasting the Future Distribution of the Caspian Whip Snake

The clamping results for all future predictions show that the climate conditions in the training region were similar to those in the projected area, across emission scenarios and time frames. The only regions where the climates were different are located in the northeastern Europe, a region in which Caspian whip snake has not been recorded. Thus, we assumed that the models would be reliably transferred to future climates since novel climatic conditions were not identified in the species’ geographic range.

**Figure 2:**
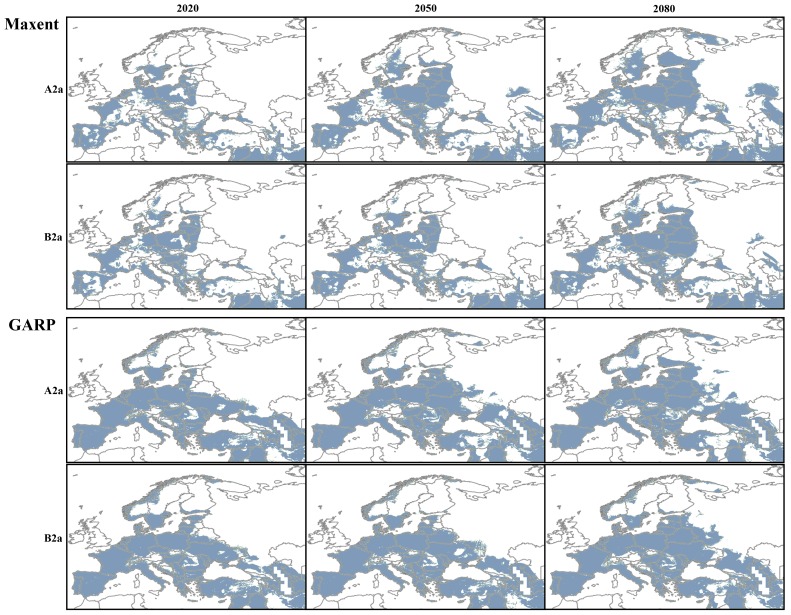
Future climatically suitable ranges for the Caspian whip snake, *Dolichophis caspius*.

The agreement between future projections produced by GARP using the liberal (A2a) and conservative (B2a) emission scenarios was high (>90% congruence between the number of pixels predicted present), for each of the three time periods studied (2020, 2050 and 2080). On the other hand, Maxent projections were more divergent between climate scenarios: over 60% congruence for 2050 and 2080 and 80% for 2020 (Table 2 and Fig. 2). The models produced by Maxent predicted a doubling of suitable climate area from the present to future projections under both climate scenarios and suitable regions extended to areas presently unsuitable for *D. caspius*.

**Table 2:** Percent of suitable niche overlap between the two emission scenarios (A2a and B2a) for the studied time periods (2020, 2050, 2080).

*Algorithm*	*2020*	*2050*	*2080*
Maxent	81.85	60.19	66.29
GARP	98.01	93.92	96.07

The models produced by GARP predicted a more conservative change of the climatically suitable areas, as future projections were more similar to present predictions, adding 10.21% more suitable space by 2080 under scenario A2a and 8.37% more suitable space under scenario B2a (Table 3). The visual analysis of the effects of climate change on *D. caspius* potential distribution using both modeling methods (GARP and Maxent) and both emission scenarios (A2a and B2a) suggests an increase in climatic suitability in the currently known distributional range and also a gradual geographic expansion of the climatic niche of the species farther north. The most significant changes in terms of expansion of suitable climates for *D. caspius* can be observed in the northern part of its range, especially in Poland, Ukraine, the Baltic states, southern and western Russia, and around Caspian Sea (figure 2).

**Table 3:** Percent of climatically suitable areas available for the Caspian whip snake of the total projected space.

		*Future climate scenarios*					
	Present	A2a			B2a		
**Algorithm**		**2020**	**2050**	**2080**	**2020**	**2050**	**2080**
**Maxent**	20.24	24.99	33.54	42.18	25.78	27.65	35.07
**GARP**	36.85	45.63	46.30	47.06	43.49	43.49	45.22

However, while there was consensus between algorithms regarding the northern expansion of the geographic range of favorable conditions, the models generated by GARP under both emission scenarios predicted a small and progressive loss of suitable areas from the species’ southern distribution limit, in Turkey, Lebanon, and Syria.

### Maximum Dispersal Range Analysis

The cost distance analysis of the three dispersal scenarios produced different results for the climate projections obtained with the two modeling algorithms (Maxent and GARP) that required different minimum cost distance thresholds to include species’ known presences.

In the case of the permissive scenario (S1), the minimum distance threshold that connected all species’ known distribution points was 5.26% of the original rasters resulted from the cost distance analysis using Maxent and the liberal A2a emission scenario, and 5.53% using the conservative B2a emission scenario. For GARP, the distance thresholds were 4.65% (A2a) and 4.87% (B2a). For the restrictive scenario (S2), the distance thresholds were 6.84% (A2a) and 7.11% (B2a) using Maxent predictions and 5.24% (A2a) and 5.53% (B2a) using GARP predictions. Finally, in the case of the balanced scenario (S3), distance thresholds were 9% using the liberal emission scenario (A2a) and 8% using the conservative emission scenario (B2a) for Maxent potential distributions, and 4% for both climatic scenarios for GARP potential distributions. This suggests a better ability of the MDRs based on GARP models output to delineate a single area that includes all points used in analysis.

Using the S1 dispersal scenario, the spatial congruence between the MDRs based on GARP was much higher (97%) than in the case of Maxent (88%), whereas using the S2 dispersal scenario the congruence was slightly higher for Maxent (98%) than GARP (96%) predictions. In the balanced S3 scenario, the spatial congruence between the MDR for the two emission scenarios was again higher for the analysis based on GARP outputs (99.16%) than the one based on Maxent outputs (92.67%).

The area defined as MDR using the Maxent projections on the liberal A2a scenario represented 20.4% of the total area of the projected model and 23.27% of the conservative B2a scenario in the case of the permissive scenario (S1, Table 4). The MDRs based on the GARP models for the two scenarios were similar, with 22.35% of the total area of the projected model using the A2a scenario and 23.06% using the B2a scenario (Table 4). In the case of the restrictive scenario (S2), the area defined as MDR using Maxent niche projections were 20.33% using the A2a emissions scenario and 20.81% with the B2a emissions scenario (Table 4). The MDRs based on the GARP projections represented approximately19% (18.72% for A2a and 19.43% for B2a) of the total projected area under both emission scenarios (Table 4). The MDRs from the balanced model (S3) represented 30.34% of the total area of the projected model using the A2a scenario and 28.12% of the conservative B2a scenario projections. The MDRs based on the GARP models for the two emission scenarios featured 21.73% of the total area of the projected model using the A2a scenario and 21.54% using the B2a scenario (Table 4).

**Table 4:** Percent of the area predicted accessible for *D. caspius* of the total projected space in the context of global warming based on two climate change emission scenarios (A2a and B2a) and the three scenarios of Maximum Dispersal Range (MDR).

Dispersal scenario	*MDR*	*Future climate scenarios*	
		A2a	B2a
**S1**	**Maxent based MDR**	20.4	23.27
	**GARP based MDR**	22.35	23.06
**S2**	**Maxent based MDR**	20.33	20.81
	**GARP based MDR**	18.72	19.43
**S3**	**Maxent based MDR**	30.34	28.12
	**GARP based MDR**	21.73	21.54

**Figure 3:**
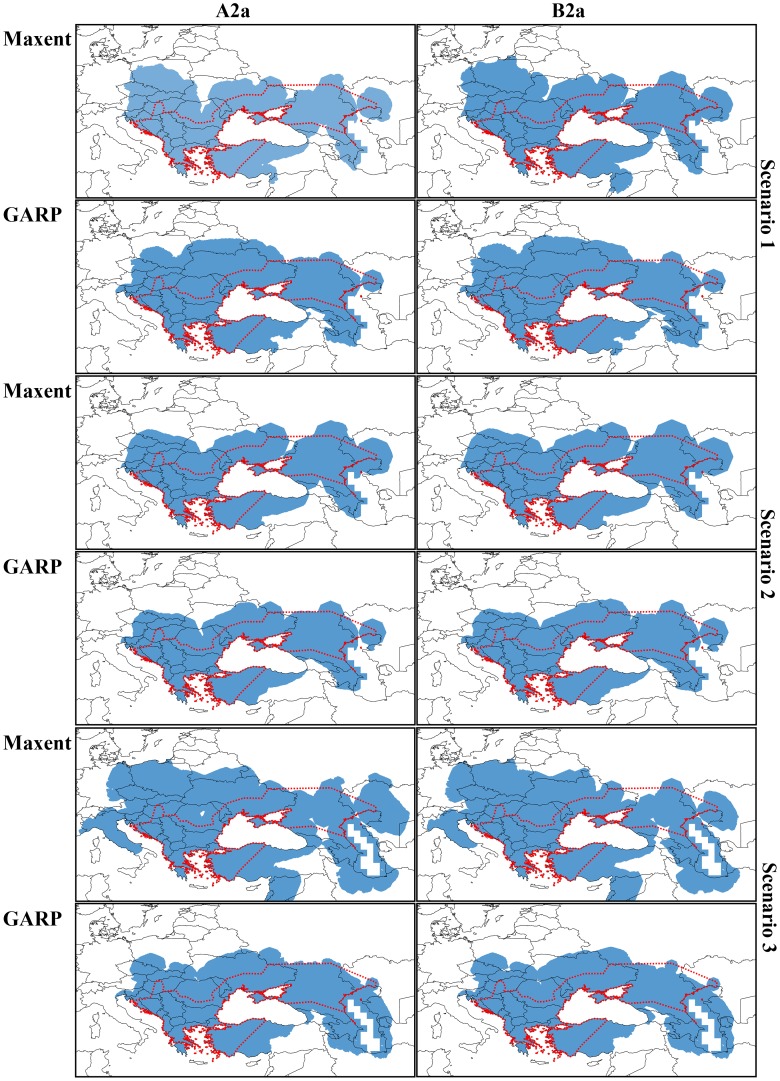
Maximum dispersal range estimates (blue) using Maxent and GARP future potential distributions (A2a – liberal scenario and B2a – conservative scenario) and three dispersal scenarios (S1 - permissive scenario, S2 - restrictive scenario, S3 - balanced scenario). The known species’ range is shown in red dotted line.

Even though the degree of congruence between MDRs within each algorithm (Maxent and GARP) and between climatic scenarios was >90%, slight differences existed across algorithms and scenarios. The MDRs based on GARP predictions showed many more dispersal possibilities for the Caspian whip snake when the analyses were based on the permissive S1 scenario, the MDR covering 98% (A2a) and 97.55% (B2A) of the current distribution range, while the MDRs based on Maxent covered 95.67% (A2a) and 97.20% (B2a) of the current distribution range (Figure 3). By using S2, the MDRs identified similar dispersal options, the MDRs based on GARP covering 98% (A2a) and 97.90% of the current distribution range and the Maxent-based MDRs covered 98.55% (A2a) and 98.89% (B2a) of the Caspian whip snake’s current distribution (Figure 3). In S3 the MDRs based on the Maxent predictions indicated many dispersal options, even more so when taking into account the liberal A2a model, with a 97.61% overlap with the current distribution range of the Caspian whip snake, in contrast to the conservative B2a model with a 94.69% overlap with the current distribution (Figure 3). The MDRs based on GARP predictions overlapped with the current distribution of *D. caspius* in 98.20% of the area for the liberal A2a climatic scenario and 96.09% for the conservative B2a climatic scenario (Figure 3).

In general, using the S1 dispersal scenario, the MDRs based on GARP predict dispersal options for the Caspian whip snake into Austria, Slovakia, the Czech Republic, Poland, Ukraine, Belarus, and Russia towards the north, but also further into Turkey in the southeast and into Georgia, Armenia, Azerbaijan, and Iran in the south. In the case of the MDRs based on the Maxent models, dispersal options are identified as far north as northern Poland and to the west all the way into eastern Germany, but unlike the GARP-based predictions, Ukraine and Belarus are shown only as limited routes for dispersal. As in the case of the GARP-based MDRs, to the south Georgia, Armenia, Azerbaijan, and Iran are presented as likely dispersal routes, but unlike the GARP-based MDRs, the outputs based on Maxent identify a sizable part of Syria and Lebanon as possible dispersal options.

Using the restrictive scenario (S2), the consensus was greater across modeling algorithms and emission scenarios (A2a & B2a), all identifying Slovenia, Austria, the Czech Republic, southern Poland, Ukraine, and Russia as likely dispersal routes towards the north, Georgia, Azerbaijan, and northwestern Iran in the south, and Turkey in the south-east.

In the case of the balanced scenario (S3), as a general agreement between MDRs produced based on Maxent and GARP across climate change emission scenarios, we identified favorable corridors and a relatively low resistance of the landscape for *D. caspius* to disperse to northwestern parts of its range to the Czech Republic, Slovakia, Poland, and Slovenia, assuming no significant changes of the human footprint or ecoregion distribution in the future. Other important areas where *D. caspius* would have the possibility to expand its range are Anatolia and the Caucasus countries, especially Georgia and Azerbaijan. On the other hand, the models predicted reductions in the northern and eastern parts of the species’ current range. However, according to our MDRs, large parts of Ukraine, Romania, and Moldova will be climatically suitable and accessible by the Caspian whip snake.

## Discussion

Although Maxent model had higher partial ROC AUC values, GARP model performed better in the two other evaluation methods, omission error (no presences predicted absent) and herpetologist expert opinion. These results may be a consequence of the basic differences between GARP and Maxent, as the former tends to produce models with higher commission error than the latter, in other words predict suitable broader areas [88], therefore the herpetologist expert opinion would be in agreement with the models generated by GARP.

Our models identified several environmental variables that had high contribution to generating the potential distribution prediction of *D. caspius* that recovered the current known range as well as identified other, geographically adjacent, climatically suitable areas. As it is the case with all reptiles in general, the Caspian whip snake’s large-scale distribution is mainly environmentally dependent, due to its physiological characteristics. Of all 19 variables initially used in the modeling process, the most important ones that we based our final models on, and that best explained the environmental requirements of the species, were four temperature-derived variables and one precipitation-derived variable. Our findings are in agreement with the previous research published on this species that characterized it as xerophilous [66]–[68]. The species is known to tolerate high temperatures and long dry periods (up to several months) [66], but it cannot tolerate low temperatures during winter, the latter being considered a limiting factor of its distribution, frequently indicated in the herpetological literature [66]–[68]. This limiting factor was represented in our models by two variables, the mean temperature of coldest quarter and the minimum temperature of the coldest month, which limited the species’ distribution northwards of 50° latitude in the Eastern European Plains. The critical 50° latitude is also indicated in literature as the northernmost limit of the species’ distribution but here we identify two climate factors that may explain the observed northern distributional limit. On the other hand, *D. caspius* is not able to live in desert and semi-desert environments and this also was captured by our models which indicated as limiting factor the mean diurnal range variable in Near East, Middle East, and Central and Southern Iran due to the very high temperatures during the warm period, which are frequent in the area. In contrast, in most of the Mediterranean basin and Central and Western Europe, *D. caspius* models had no limitations, thus probably other, non-climate variables such as dispersal ability of the species and landscape features play an important role in shaping its distribution.

The projection of models onto future climatic conditions, for both algorithms used and both emissions scenarios (the liberal A2a and the conservative B2a), predicted similar trends in distributional shifts of the *D. caspius*. According to these models, as a result of global warming, suitable climatic conditions for *D. caspius* will be present in geographic areas north of its current range, especially in the Central and Western Europe, but also in the Eastern European Plains. The latter region, under current conditions, is considered highly unsuitable in the literature and by our models built on current climatic data. The projected climate changes may present the opportunity for *D. caspius* to migrate to new regions northwards of the climate-driven 50° latitude barrier.

The global warming process was estimated at a rate of 0.2°C per decade for the next two decades in most global climate models [80]. At this accelerated rate of warming, the predicted effects in most cases are range contractions or, in extreme situations, complete relocation to a new geographic area where the climate would become favorable. Studies on the effects of global warming on reptile species have generally predicted negative consequences [28]–[30], [95], [96]. In contrast, our models generated using Maxent and GARP show that the climatic space available for *D. caspius* will expand geographically, especially beyond the northern distribution limit.

While expansion is indicated by both algorithms across both climate change scenarios, this geographic expansion of favorable climatic conditions for *D. caspius* does not automatically infer actual distribution expansion. As numerous studies have shown, while the distribution of a species seems to be influenced mostly by climatic conditions at large scales, at finer scales additional factors become essential [37], [82], [97], [98], such as landscape features, ecological communities, predator-prey interactions, and anthropogenic pressures [99], [100]. Moreover, as these factors are responsible for the existence of a species at local scales, the large-scale distribution of the species will depend on the existence of favorable climatic conditions plus the dispersal ability. The MDR analysis performed here reveals precisely the importance of dispersal: while novel areas with suitable conditions will arise for *D. caspius* outside its current range, its low dispersal ability will impede the colonization of the distant areas with newly suitable climates. Thus, MDRs are in agreement with current views regarding the effects of global climate change on amphibians and reptiles.

### Limitations of the Maximum Dispersal Range Analysis

The MDRs developed in this study estimate the dispersal range by associating a difficulty score to environmental or physical features that individuals may encounter in their migration paths. While these simulations too rely on certain assumptions, such as unchanging human land use patterns and ecological communities, our opinion is that this method provides additional, essential information to refine views on the impact of global warming on species’ distribution.

Using the MDR analysis, we expand our ability to assess the impacts of global warming on species’ distributions. However, to use this technique, researchers are expected to have advanced knowledge regarding the ecology, habitat requirements, and dispersal potential of the studied species, information that is crucial to creating the cost raster for the MDR analysis. Nevertheless, this method cannot be standardized to all species due to differences in dispersal ability or lack of sufficient knowledge of a certain species’ ecology. Thus, we recommend careful selection and prioritization of the parameters for the cost analysis, on a species by species basis.

Another limitation to the method is the omission of adaptation capability of individual species. While certain species (e.g., *Elaphe sauromates, Zamenis longissimus*) are known for their sensitivity to environmental changes, especially the human-induced ones [101], [102], other species (e.g., *Bufo viridis*, *Lacerta viridis*, *Natrix natrix*) exhibit ecological plasticity [103], [104]. Thus generating the MDRs and inferring the results need to be based on knowledge of the species’ biology.

## Supporting Information

Supporting Information S1 **Georeferenced occurrence points for** ***Dolichophis caspius*** **.**
(ZIP)

Supporting Information S2 **Maps indicating areas in which used climatic variables were most dissimilar between present and future climatic conditions.** Each color corresponds to a variable that in the future will be very dissimilar compared with current conditions. Red corresponds to minimum temperature of the coldest month, Light blue corresponds to mean temperature of the coldest quarter, Yellow corresponds to temperature isothermality, Dark blue corresponds to mean diurnal range, and Purple corresponds to precipitation of driest month.
(TIF)

Supporting Information S3 **Maps corresponding to each cost raster used to predict future dispersal (Maximum Dispersal Range) in context of the climate change predictions.** Colors represent an gradient of cost, from blue (lowest cost) to red (highest cost).
(TIF)

Supporting Information S4 **Environmental variables used in the MDR Analysis, reclassification ranges, “cost” values assigned and sources for the datasets.**
(DOCX)

Supporting Information S5 **Additional information regarding environmental data acquisition.**
(DOCX)

Supporting Information S6 **Additional information regarding the creation of the “cost” layer for the MDR Analysis.**
(DOCX)
