# Supplementary figures and images for: Refining Climate Change Projections for Organisms with Low Dispersal Abilities: A Case Study of the Caspian Whip Snake
Source: PLoS One. 2014 Mar 26;9(3):e91994. doi: 10.1371/journal.pone.0091994 (PMC3966777; doi:10.1371/journal.pone.0091994)

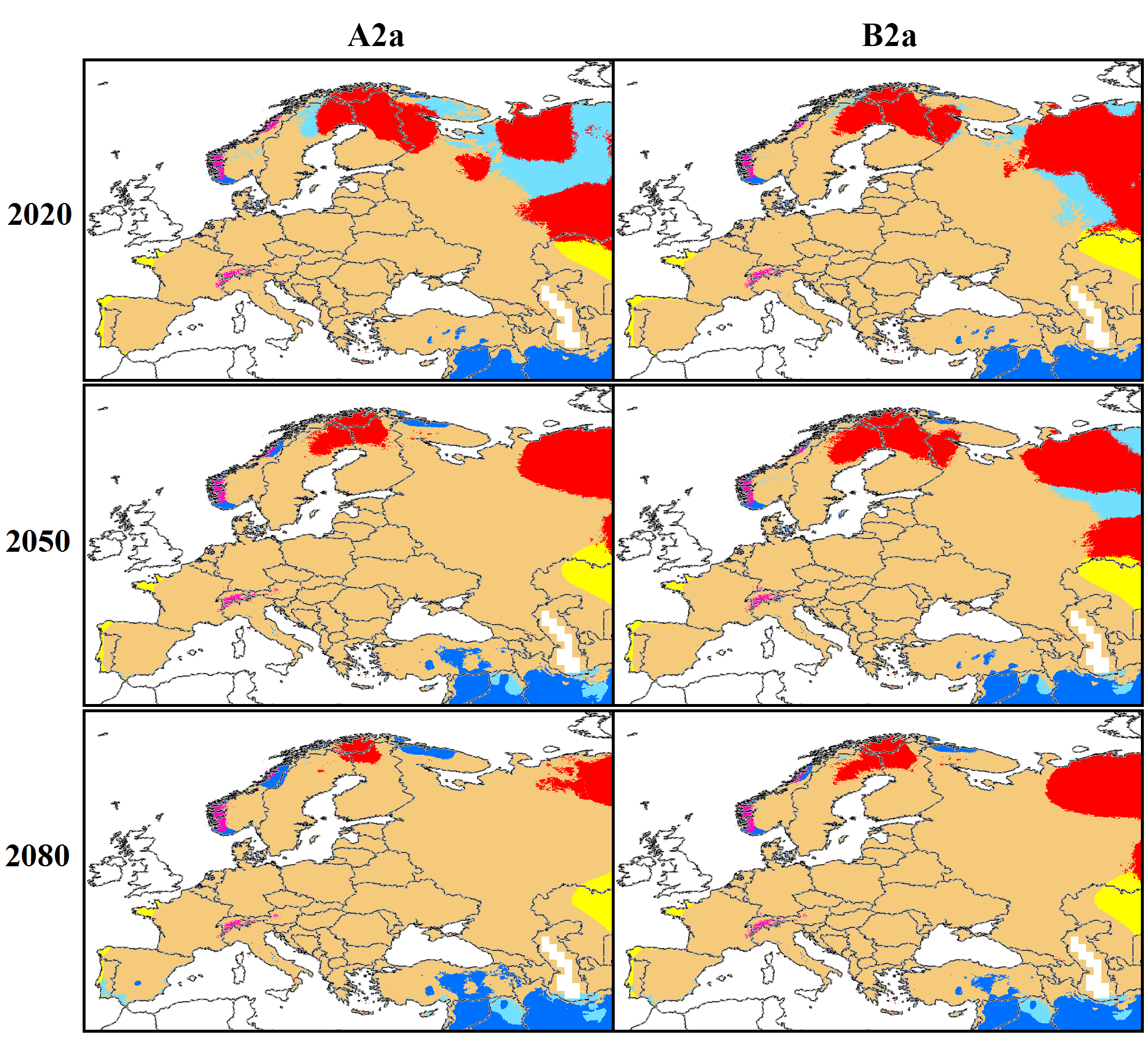

Supplement: Supporting Information S2 — Maps indicating areas in which used climatic variables were most dissimilar between present and future climatic conditions. Each color corresponds to a variable that in the future will be very dissimilar compared with current conditions. Red corresponds to minimum temperature of the coldest month, Light blue corresponds to mean temperature of the coldest quarter, Yellow corresponds to temperature isothermality, Dark blue corresponds to mean diurnal range, and Purple corresponds to precipitation of driest month. (TIF) [file pone.0091994.s002.tif]

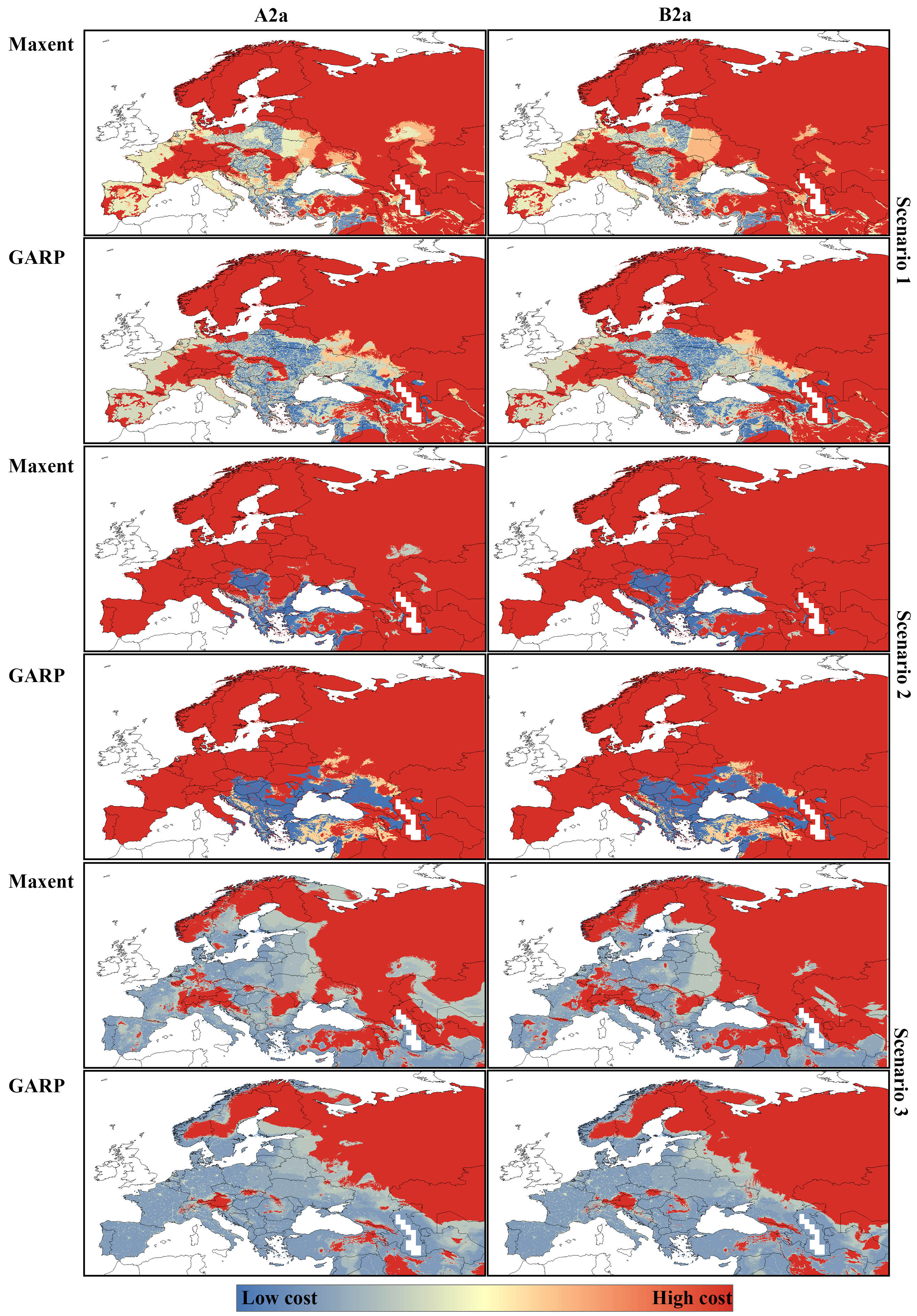

Supplement: Supporting Information S3 — Maps corresponding to each cost raster used to predict future dispersal (Maximum Dispersal Range) in context of the climate change predictions. Colors represent an gradient of cost, from blue (lowest cost) to red (highest cost). (TIF) [file pone.0091994.s003.tif]
