# Supplementary material for: Refining Climate Change Projections for Organisms with Low Dispersal Abilities: A Case Study of the Caspian Whip Snake
Source: PLoS One. 2014 Mar 26;9(3):e91994. doi: 10.1371/journal.pone.0091994 (PMC3966777; doi:10.1371/journal.pone.0091994)
Supplement: Supporting Information S4 — Environmental variables used in the MDR Analysis, reclassification ranges, “cost” values assigned and sources for the datasets. (DOCX) [file pone.0091994.s004.docx]

# S4. ENVIRONMENTAL VARIABLES USED IN THE MDR ANALYSIS, RECLASSIFICATION RANGES, "COST" VALUES ASSIGNED AND SOURCES FOR THE DATASETS

S4-1. Variables used, reclassification ranges and reclassified cost values for the *permissive scenario* *(S1)*

| **Environmental variable** | **Category name and/or range** | **Reclassified cost value** | **Source** |
| --- | --- | --- | --- |
| **4.5 Km DEM** | **Altitude (m)** |  | www.worldclim.org |
|  | -100 - 600 | 1 |  |
|  | 601 - 1000 | 2 |  |
|  | 1001 - 1700 | 5 |  |
|  | >1701 | 10 |  |
| **Large rivers** | **Presence/absence** | **Reclassified cost value** | **Source** |
|  | 0 (absent) | 0 | ArcGIS data |
|  | 1 (present) | 10 |  |
| **Human footprint** | **Raster value as degree of human habitation** | **Reclassified cost value** | **Source** |
|  | 0-30 (natural and semi-natural areas) | 1 | sedac.ciesin.columbia.edu |
|  | 31 - 50 (Villages and other areas with low human presence) | 5 |  |
|  | 51 - 75 (Towns, small cities and other areas with sustained human habitation) | 7 |  |
|  | 76 - 100 (Urban centers and densely populated areas ) | 10 |  |
| **Climatic Models A2a & B2a** | **Model classes** | **Reclassified cost value** | **Source** |
|  | 0 | 10 | ENMs generated using MaxEnt and GARP |
|  | 1 | 7 |  |
|  | 2 | 5 |  |
|  | 3 | 1 |  |
|  | 4 | 1 |  |
| **WWF Ecoregions** | **Ecoregion name** | **Reclassified cost value** | **Source** |
| **Ecoregion code** |  |  |  |
| 81201 | Aegean & Western Turkey sclerophyllous and mixed forests | 1 | worldwildlife.org/biomes |
| 80404 | Balkan mixed forests | 1 |  |
| 80814 | Pontic steppe | 1 |  |
| 80431 | Pannonian mixed forests | 1 |  |
| 80419 | East European forest steppe | 1 |  |
| 81210 | Illyrian deciduous forests | 1 |  |
| 80435 | Rodope montane mixed forests | 1 |  |
| 81217 | Pindus Mountains mixed forests | 1 |  |
| 80422 | Euxine-Colchic broadleaf forests | 1 |  |
| 81202 | Anatolian conifer and deciduous mixed forests | 1 |  |
| 81308 | Caspian lowland desert | 1 |  |
| 81222 | Tyrrhenian-Adriatic Sclerophyllous and mixed forests | 1 |  |
| 80410 | Central Anatolian steppe and woodlands | 1 |  |
| 80416 | Crimean Submediterranean forest complex | 1 |  |
| 80418 | Dinaric Mountains mixed forests | 2 |  |
| 81220 | Southern Anatolian montane conifer and deciduous forests | 2 |  |
| 81305 | Azerbaijan shrub desert and steppe | 2 |  |
| -9998 | Lake | 10 |  |
| 80401 | Appenine deciduous montane forests | 5 |  |
| 80402 | Atlantic mixed forests | 5 |  |
| 80405 | Baltic mixed forests | 10 |  |
| 80406 | Cantabrian mixed forests | 5 |  |
| 80407 | Caspian Hyrcanian mixed forests | 5 |  |
| 80408 | Caucasus mixed forests | 10 |  |
| 80409 | Celtic broadleaf forests | 5 |  |
| 80412 | Central European mixed forests | 2 |  |
| 80420 | Eastern Anatolian deciduous forests | 2 |  |
| 80429 | North Atlantic moist mixed forests | 10 |  |
| 80432 | Po Basin mixed forests | 5 |  |
| 80433 | Pyrenees conifer and mixed forests | 10 |  |
| 80436 | Sarmatic mixed forests | 10 |  |
| 80444 | Western Siberian hemiboreal forests | 10 |  |
| 80445 | Western European broadleaf forests | 10 |  |
| 80446 | Zagros Mountains forest steppe | 5 |  |
| 80501 | Alps conifer and mixed forests | 10 |  |
| 80504 | Carpathian montane forests | 10 |  |
| 80507 | Elburz Range forest steppe | 5 |  |
| 80515 | Northern Anatolian conifer and deciduous forests | 2 |  |
| 80520 | Scandinavian coastal conifer forests | 10 |  |
| 80608 | Scandinavian and Russian taiga | 10 |  |
| 80610 | Ural montane forests and tundra | 10 |  |
| 80611 | West Siberian taiga | 10 |  |
| 80801 | Alai-Western Tian Shan steppe | 5 |  |
| 80803 | Central Anatolian steppe | 2 |  |
| 80805 | Eastern Anatolian montane steppe | 2 |  |
| 80809 | Kazakh forest steppe | 5 |  |
| 80810 | Kazakh steppe | 2 |  |
| 80812 | Middle East steppe | 2 |  |
| 80906 | Tigris-Euphrates alluvial salt marsh | 5 |  |
| 81004 | Ghorat-Hazarajat alpine meadow | 10 |  |
| 81008 | Kopet Dag woodlands and forest steppe | 5 |  |
| 81009 | Kuh Rud and Eastern Iran montane woodlands | 5 |  |
| 81106 | Kola Peninsula tundra | 10 |  |
| 81108 | Northwest Russian-Novaya Zemlya tundra | 10 |  |
| 81110 | Scandinavian Montane Birch forest and grasslands | 10 |  |
| 81114 | Yamal-Gydan tundra | 10 |  |
| 81205 | Crete Mediterranean forests | 2 |  |
| 81206 | Cyprus Mediterranean forests | 2 |  |
| 81207 | Eastern Mediterranean conifer-sclerophyllous-broadleaf forests | 2 |  |
| 81208 | Iberian conifer forests | 10 |  |
| 81209 | Iberian sclerophyllous and semi-deciduous forests | 5 |  |
| 81211 | Italian sclerophyllous and semi-deciduous forests | 5 |  |
| 81215 | Northeastern Spain & Southern France Mediterranean forests | 5 |  |
| 81216 | Northwest Iberian montane forests | 10 |  |
| 81218 | South Appenine mixed montane forests | 5 |  |
| 81219 | Southeastern Iberian shrubs and woodlands | 10 |  |
| 81221 | Southwest Iberian Mediterranean sclerophyllous and mixed forests | 10 |  |
| 81301 | Afghan Mountains semi-desert | 5 |  |
| 81303 | Arabian Desert and East Sahero-Arabian xeric shrublands | 10 |  |
| 81306 | Badghyz and Karabil semi-desert | 10 |  |
| 81309 | Central Afghan Mountains xeric woodlands | 5 |  |
| 81310 | Central Asian northern desert | 10 |  |
| 81311 | Central Asian riparian woodlands | 5 |  |
| 81312 | Central Asian southern desert | 10 |  |
| 81313 | Central Persian desert basins | 10 |  |
| 81318 | Kazakh semi-desert | 10 |  |
| 81319 | Kopet Dag semi-desert | 10 |  |
| 81320 | Mesopotamian shrub desert | 10 |  |
| 81322 | Paropamisus xeric woodlands | 5 |  |
| 81326 | Registan-North Pakistan sandy desert | 10 |  |
| 81328 | South Iran Nubo-Sindian desert and semi-desert | 10 |  |

**Table S4-2. Variables used, reclassification ranges and reclassified cost values for the *restrictive scenario* *(S2)***

| **Environmental variable** | **Category name and/or range** | **Reclassified cost value** | **Source** |
| --- | --- | --- | --- |
| **4.5 Km DEM** | **Altitude (m)** |  | www.worldclim.org |
|  | -100 - 600 | 1 |  |
|  | 601 - 1000 | 2 |  |
|  | 1001 - 2500 | 8 |  |
|  | >2500 | 10 |  |
| **Large rivers** | **Presence/absence** | **Reclassified cost value** | **Source** |
|  | 0 (absent) | 0 | ArcGIS data |
|  | 1 (present) | 10 |  |
| **Human footprint** | **Raster value as degree of human habitation** | **Reclassified cost value** | **Source** |
|  | 0 - 15 (Natural areas) | 1 | sedac.ciesin.columbia.edu |
|  | 16 - 30 (Agricultural and semi-natural areas) | 2 |  |
|  | 31 - 50 (Villages and other areas with low human presence) | 5 |  |
|  | 51-100 (All urban areas) | 10 |  |
| **Climatic Models A2a & B2a** | **Model classes** | **Reclassified cost value** | **Source** |
|  | 0 | 10 | ENMs generated using MaxEnt and GARP |
|  | 1 | 10 |  |
|  | 2 | 8 |  |
|  | 3 | 5 |  |
|  | 4 | 1 |  |
| **WWF Ecoregions** | **Ecoregion name** | **Reclassified cost value** | **Source** |
| **Ecoregion code** |  |  |  |
| 81201 | Aegean & Western Turkey sclerophyllous and mixed forests | 1 | worldwildlife.org/biomes |
| 80404 | Balkan mixed forests | 1 |  |
| 80814 | Pontic steppe | 1 |  |
| 80431 | Pannonian mixed forests | 2 |  |
| 80419 | East European forest steppe | 2 |  |
| 81210 | Illyrian deciduous forests | 1 |  |
| 80435 | Rodope montane mixed forests | 1 |  |
| 81217 | Pindus Mountains mixed forests | 2 |  |
| 80422 | Euxine-Colchic broadleaf forests | 2 |  |
| 81202 | Anatolian conifer and deciduous mixed forests | 2 |  |
| 81308 | Caspian lowland desert | 2 |  |
| 81222 | Tyrrhenian-Adriatic Sclerophyllous and mixed forests | 2 |  |
| 80410 | Central Anatolian steppe and woodlands | 2 |  |
| 80416 | Crimean Submediterranean forest complex | 1 |  |
| 80418 | Dinaric Mountains mixed forests | 5 |  |
| 81220 | Southern Anatolian montane conifer and deciduous forests | 5 |  |
| 81305 | Azerbaijan shrub desert and steppe | 5 |  |
| -9998 | Lake | 10 |  |
| 80401 | Appenine deciduous montane forests | 10 |  |
| 80402 | Atlantic mixed forests | 10 |  |
| 80405 | Baltic mixed forests | 10 |  |
| 80406 | Cantabrian mixed forests | 10 |  |
| 80407 | Caspian Hyrcanian mixed forests | 10 |  |
| 80408 | Caucasus mixed forests | 10 |  |
| 80409 | Celtic broadleaf forests | 10 |  |
| 80412 | Central European mixed forests | 10 |  |
| 80420 | Eastern Anatolian deciduous forests | 10 |  |
| 80429 | North Atlantic moist mixed forests | 10 |  |
| 80432 | Po Basin mixed forests | 10 |  |
| 80433 | Pyrenees conifer and mixed forests | 10 |  |
| 80436 | Sarmatic mixed forests | 10 |  |
| 80444 | Western Siberian hemiboreal forests | 10 |  |
| 80445 | Western European broadleaf forests | 10 |  |
| 80446 | Zagros Mountains forest steppe | 10 |  |
| 80501 | Alps conifer and mixed forests | 10 |  |
| 80504 | Carpathian montane forests | 10 |  |
| 80507 | Elburz Range forest steppe | 10 |  |
| 80515 | Northern Anatolian conifer and deciduous forests | 10 |  |
| 80520 | Scandinavian coastal conifer forests | 10 |  |
| 80608 | Scandinavian and Russian taiga | 10 |  |
| 80610 | Ural montane forests and tundra | 10 |  |
| 80611 | West Siberian taiga | 10 |  |
| 80801 | Alai-Western Tian Shan steppe | 10 |  |
| 80803 | Central Anatolian steppe | 10 |  |
| 80805 | Eastern Anatolian montane steppe | 10 |  |
| 80809 | Kazakh forest steppe | 10 |  |
| 80810 | Kazakh steppe | 10 |  |
| 80812 | Middle East steppe | 10 |  |
| 80906 | Tigris-Euphrates alluvial salt marsh | 10 |  |
| 81004 | Ghorat-Hazarajat alpine meadow | 10 |  |
| 81008 | Kopet Dag woodlands and forest steppe | 10 |  |
| 81009 | Kuh Rud and Eastern Iran montane woodlands | 10 |  |
| 81106 | Kola Peninsula tundra | 10 |  |
| 81108 | Northwest Russian-Novaya Zemlya tundra | 10 |  |
| 81110 | Scandinavian Montane Birch forest and grasslands | 10 |  |
| 81114 | Yamal-Gydan tundra | 10 |  |
| 81205 | Crete Mediterranean forests | 10 |  |
| 81206 | Cyprus Mediterranean forests | 10 |  |
| 81207 | Eastern Mediterranean conifer-sclerophyllous-broadleaf forests | 10 |  |
| 81208 | Iberian conifer forests | 10 |  |
| 81209 | Iberian sclerophyllous and semi-deciduous forests | 10 |  |
| 81211 | Italian sclerophyllous and semi-deciduous forests | 10 |  |
| 81215 | Northeastern Spain & Southern France Mediterranean forests | 10 |  |
| 81216 | Northwest Iberian montane forests | 10 |  |
| 81218 | South Appenine mixed montane forests | 10 |  |
| 81219 | Southeastern Iberian shrubs and woodlands | 10 |  |
| 81221 | Southwest Iberian Mediterranean sclerophyllous and mixed forests | 10 |  |
| 81301 | Afghan Mountains semi-desert | 10 |  |
| 81303 | Arabian Desert and East Sahero-Arabian xeric shrublands | 10 |  |
| 81306 | Badghyz and Karabil semi-desert | 10 |  |
| 81309 | Central Afghan Mountains xeric woodlands | 10 |  |
| 81310 | Central Asian northern desert | 10 |  |
| 81311 | Central Asian riparian woodlands | 10 |  |
| 81312 | Central Asian southern desert | 10 |  |
| 81313 | Central Persian desert basins | 10 |  |
| 81318 | Kazakh semi-desert | 10 |  |
| 81319 | Kopet Dag semi-desert | 10 |  |
| 81320 | Mesopotamian shrub desert | 10 |  |
| 81322 | Paropamisus xeric woodlands | 10 |  |
| 81326 | Registan-North Pakistan sandy desert | 10 |  |
| 81328 | South Iran Nubo-Sindian desert and semi-desert | 10 |  |

**Table S4-3. Variables used, reclassification ranges and reclassified cost values for the *balanced scenario (S3)***

| **Environmental variable** | **Category name and/or range** | **Reclassified cost value** | **Source** |
| --- | --- | --- | --- |
| **4.5 Km DEM** | **Altitude (m)** |  | www.worldclim.org |
|  | -100 - 300 | 35 |  |
|  | 301 - 600 | 37 |  |
|  | 601 - 1000 | 60 |  |
|  | 1001 - 1700 | 65 |  |
|  | 1701 - 2500 | 250 |  |
|  | > 2500 | 1000 |  |
| **Large rivers** | **Presence/absence** | **Reclassified cost value** | **Source** |
|  | 0 (absent) | 0 | ArcGIS data |
|  | 1 (present) | 750 |  |
| **Human footprint** | **Raster value as degree of human habitation** | **Reclassified cost value** | **Source** |
|  | 0 - 15 (Natural areas) | 15 | sedac.ciesin.columbia.edu |
|  | 16 - 30 (Agricultural and semi-natural areas) | 25 |  |
|  | 31 - 50 (Villages and other areas with low human presence) | 35 |  |
|  | 51 - 75 (Towns, small cities and other areas with sustained human habitation) | 300 |  |
|  | 76 - 100 (Urban centers and densely populated areas ) | 950 |  |
| **Climatic Models A2a & B2a** | **Model classes** | **Reclassified cost value** | **Source** |
|  | 0 | 1000000 | ENMs generated using MaxEnt and GARP |
|  | 1 | 130 |  |
|  | 2 | 120 |  |
|  | 3 | 110 |  |
|  | 4 | 100 |  |
| **WWF Ecoregions** | **Ecoregion name** | **Reclassified cost value** | **Source** |
| **Ecoregion code** |  |  |  |
| 81201 | Aegean & Western Turkey sclerophyllous and mixed forests | 10 | worldwildlife.org/biomes |
| 80404 | Balkan mixed forests | 10 |  |
| 80814 | Pontic steppe | 10 |  |
| 80431 | Pannonian mixed forests | 20 |  |
| 80419 | East European forest steppe | 20 |  |
| 81210 | Illyrian deciduous forests | 10 |  |
| 80435 | Rodope montane mixed forests | 10 |  |
| 81217 | Pindus Mountains mixed forests | 20 |  |
| 80422 | Euxine-Colchic broadleaf forests | 20 |  |
| 81202 | Anatolian conifer and deciduous mixed forests | 30 |  |
| 81308 | Caspian lowland desert | 30 |  |
| 81222 | Tyrrhenian-Adriatic Sclerophyllous and mixed forests | 30 |  |
| 80410 | Central Anatolian steppe and woodlands | 30 |  |
| 80416 | Crimean Submediterranean forest complex | 10 |  |
| 80418 | Dinaric Mountains mixed forests | 50 |  |
| 81220 | Southern Anatolian montane conifer and deciduous forests | 50 |  |
| 81305 | Azerbaijan shrub desert and steppe | 50 |  |
| -9998 | Lake | 750 |  |
| 80401 | Appenine deciduous montane forests | 200 |  |
| 80402 | Atlantic mixed forests | 500 |  |
| 80405 | Baltic mixed forests | 750 |  |
| 80406 | Cantabrian mixed forests | 500 |  |
| 80407 | Caspian Hyrcanian mixed forests | 200 |  |
| 80408 | Caucasus mixed forests | 900 |  |
| 80409 | Celtic broadleaf forests | 100 |  |
| 80412 | Central European mixed forests | 75 |  |
| 80420 | Eastern Anatolian deciduous forests | 50 |  |
| 80429 | North Atlantic moist mixed forests | 900 |  |
| 80432 | Po Basin mixed forests | 500 |  |
| 80433 | Pyrenees conifer and mixed forests | 900 |  |
| 80436 | Sarmatic mixed forests | 600 |  |
| 80444 | Western Siberian hemiboreal forests | 1000 |  |
| 80445 | Western European broadleaf forests | 600 |  |
| 80446 | Zagros Mountains forest steppe | 200 |  |
| 80501 | Alps conifer and mixed forests | 750 |  |
| 80504 | Carpathian montane forests | 900 |  |
| 80507 | Elburz Range forest steppe | 200 |  |
| 80515 | Northern Anatolian conifer and deciduous forests | 50 |  |
| 80520 | Scandinavian coastal conifer forests | 1000 |  |
| 80608 | Scandinavian and Russian taiga | 1000 |  |
| 80610 | Ural montane forests and tundra | 1000 |  |
| 80611 | West Siberian taiga | 1000 |  |
| 80801 | Alai-Western Tian Shan steppe | 200 |  |
| 80803 | Central Anatolian steppe | 50 |  |
| 80805 | Eastern Anatolian montane steppe | 50 |  |
| 80809 | Kazakh forest steppe | 200 |  |
| 80810 | Kazakh steppe | 75 |  |
| 80812 | Middle East steppe | 75 |  |
| 80906 | Tigris-Euphrates alluvial salt marsh | 100 |  |
| 81004 | Ghorat-Hazarajat alpine meadow | 1000 |  |
| 81008 | Kopet Dag woodlands and forest steppe | 200 |  |
| 81009 | Kuh Rud and Eastern Iran montane woodlands | 400 |  |
| 81106 | Kola Peninsula tundra | 1000 |  |
| 81108 | Northwest Russian-Novaya Zemlya tundra | 1000 |  |
| 81110 | Scandinavian Montane Birch forest and grasslands | 1000 |  |
| 81114 | Yamal-Gydan tundra | 1000 |  |
| 81205 | Crete Mediterranean forests | 50 |  |
| 81206 | Cyprus Mediterranean forests | 50 |  |
| 81207 | Eastern Mediterranean conifer-sclerophyllous-broadleaf forests | 50 |  |
| 81208 | Iberian conifer forests | 800 |  |
| 81209 | Iberian sclerophyllous and semi-deciduous forests | 200 |  |
| 81211 | Italian sclerophyllous and semi-deciduous forests | 200 |  |
| 81215 | Northeastern Spain & Southern France Mediterranean forests | 300 |  |
| 81216 | Northwest Iberian montane forests | 900 |  |
| 81218 | South Appenine mixed montane forests | 450 |  |
| 81219 | Southeastern Iberian shrubs and woodlands | 800 |  |
| 81221 | Southwest Iberian Mediterranean sclerophyllous and mixed forests | 800 |  |
| 81301 | Afghan Mountains semi-desert | 500 |  |
| 81303 | Arabian Desert and East Sahero-Arabian xeric shrublands | 600 |  |
| 81306 | Badghyz and Karabil semi-desert | 700 |  |
| 81309 | Central Afghan Mountains xeric woodlands | 400 |  |
| 81310 | Central Asian northern desert | 1000 |  |
| 81311 | Central Asian riparian woodlands | 300 |  |
| 81312 | Central Asian southern desert | 1000 |  |
| 81313 | Central Persian desert basins | 750 |  |
| 81318 | Kazakh semi-desert | 600 |  |
| 81319 | Kopet Dag semi-desert | 600 |  |
| 81320 | Mesopotamian shrub desert | 750 |  |
| 81322 | Paropamisus xeric woodlands | 500 |  |
| 81326 | Registan-North Pakistan sandy desert | 1000 |  |
| 81328 | South Iran Nubo-Sindian desert and semi-desert | 900 |  |
