# Supplementary material for: Refining Climate Change Projections for Organisms with Low Dispersal Abilities: A Case Study of the Caspian Whip Snake
Source: PLoS One. 2014 Mar 26;9(3):e91994. doi: 10.1371/journal.pone.0091994 (PMC3966777; doi:10.1371/journal.pone.0091994)
Supplement: Supporting Information S5 — Additional information regarding environmental data acquisition. (DOCX) [file pone.0091994.s005.docx]

# S5. ADDITIONAL INFORMATION REGARDING ENVIRONMENTAL DATA ACQUISITION

In order to evaluate the dispersal ability of *Dolichophis caspius* to new areas with suitable climatic niche conditions the authors tried to take into account as many meaningful environmental variables as possible and which can be quantified into raster layers to be further used in a GIS system (in this particular case ArcGIS 10).

The final list of environmental predictors used in the analysis was composed of 6 raster files: a Digital Elevation Model (DEM), Slope, Rivers, Human Footprint, the results from both modeling algorithms (MaxEnt and GARP) using all climatic scenarios (A2a and B2a) and the Ecoregions of the World. All rasters were used as ESRI grid files.

1. **Digital Elevation Model (DEM)**. The raster file was downloaded from the Worldclim website (www.worldclim.org) at a resolution of 4.5 km (~2.5 arc-minutes) and clipped to the boundaries of the study area using ArcGIS10 (Data Management > Clip). *Dolichophis caspius* is known as a species that prefers warmer climatic conditions, inhabiting mostly steppes, forest steppes and bushy areas (Fuhn 1969) and altitude (especially highland areas) plays an important role in shaping its distribution.

2. **Rivers and large water bodies**. A shapefile with the large rivers and waterbodies of Europe was aquired from ArcGIS. In order to avoid range breaks (i.e. gaps in the pixels through which the animal can move without effort) a buffer of 500 meters was created around the rivers to simulate river width (Analysis Tools > Prximity > Buffer). Both the rivers and large water bodies polygon shapefiles were merged into one single polygon shapefile (Data Management > Merge). Further the polygon features were converted to a raster file using the Polygon to Raster function in ArcGIS 10 (Conversion > To Raster > Polygon to Raster). The resulting raster was reclassified, choosing 1 for the presence of water and 0 for the rest of the extent. *D. caspius* can easily cross small water bodies and, like all snakes, is a very good swimmer, but large rivers (ex.: Danube, Volga, Prut), natural or artificial lakes and seas can either slow dispersal rates or impede movement, isolating populations. Furthermore, crossing water bodies exposes the animals to additional dangers such as the risk of drowning (in case of large features that need crossing), injuries and predation, especially from fishes and birds.

3. **Human footprint**. The raster file for Europe and Asia was downloaded from the Center for International Earth Science Information Network website (http:// sedac.ciesin.columbia.edu) at a resolution of 1 km (~30 arc-seconds) and clipped to the area of interest using the same procedure as for the Digital Elevation Model. *D. caspius*, while often hunting in semi-natural habitats such as agricultural fields, pastures and orchards, is not often seen around human settlements. Moreover, roads are recognized as some of the most important anthropogenic modifications of the last century (Trombulack & Frissell, 2000) and their effects on amphibians and reptiles have been well documented (ex.: Andrews et al. 2008, Roe et al. 2006, Vijayakumar et al. 2001).

4. **ENMs from MaxEnt and GARP including both climatic scenarios (A2a and B2a)**. The models generated by both algorithms were converted from ASCII to ESRI grids.

5. **Ecoregions of the World**. The polygon shapefile containing the terrestrial ecoregions was downloaded from the World Wide Fund for Nature website (WWF - http://worldwildlife.org/biomes) and clipped to the region of interest using ArcGIS 10 (Analysis Toolbox > Extract > Clip). Further the polygon layer was converted to raster using the same procedure as described for the large rivers and waterbodies, using the code for the ecoregion as the value field, in order to be able to reclassify the raster. The Caspian whip snake is a relatively selective snake, inhabiting certain types of habitats, mostly within arid areas, but not desert or sub-desert regions. The Terrestrial Ecoregions of the World (TEOW) dataset groups distinct assemblages of natural communities, among which are certain units that would favor the dispersal of *D. caspius* while others, especially large forest bodies, fragmented landscapes and open habitats, would alter its migration possibilities.
