# Supplementary material for: Refining Climate Change Projections for Organisms with Low Dispersal Abilities: A Case Study of the Caspian Whip Snake
Source: PLoS One. 2014 Mar 26;9(3):e91994. doi: 10.1371/journal.pone.0091994 (PMC3966777; doi:10.1371/journal.pone.0091994)
Supplement: Supporting Information S6 — Additional information regarding the creation of the “cost” layer for the MDR Analysis. (DOCX) [file pone.0091994.s006.docx]

# S6. ADDITIONAL INFORMATION REGARDING THE CREATION OF THE "COST" LAYER FOR THE *MDR ANALYSIS*

After acquiring all the necessary environmental layers needed and converting them to ESRI grids, the next step is to reclassify the raster files and assign each interval a "cost" value. The "cost" value or "friction", as described in the paper, represents the resistance assigned to certain features in the landscape when something (in this case an animal) tries to pass through. Higher "cost" values equal higher resistance to movement.

In order to reclassify the raster files we used the Reclassify function in ArcGIS 10: Spatial Analyst > Reclass > Reclassify. The reclassification type was set to manual and the number of classes varied depending on the raster dataset. The values for each interval were entered manually.

After raster raster reclassification was complete the "cost" rasters were created using the Mosaic to New Raster command (Data Management > Raster > Mosaic to New Raster), and assigning priority to features associated with greater costs. A "cost" raster was created for each algorithm (MaxEnt & GARP) under both climatic scenarios (A2a and B2a).

Finally the MDR analysis was run using the Cost Distance function in ArcGIS 10. The animals moving through the "cost" rasters were the occurence points gathered from the literature survey. The procedure was repeated for both algorithms and all climatic projections. As no literature on home range or distances traveled is available for the Caspian whip snake, the maximum dispersal range was considered as the lowest area percentile that could cover all the distribution points recorded.
